# Target SSR-Seq: A Novel SSR Genotyping Technology Associate With Perfect SSRs in Genetic Analysis of Cucumber Varieties

**DOI:** 10.3389/fpls.2019.00531

**Published:** 2019-04-24

**Authors:** Jingjing Yang, Jian Zhang, Ruixi Han, Feng Zhang, Aijun Mao, Jiang Luo, Bobo Dong, Hui Liu, Hao Tang, Jianan Zhang, Changlong Wen

**Affiliations:** ^1^Beijing Vegetable Research Center (BVRC), Beijing Academy of Agricultural and Forestry Sciences, National Engineering Research Center for Vegetables, Beijing, China; ^2^Beijing Key Laboratory of Vegetable Germplasm Improvement, Beijing, China; ^3^Development Center of Science and Technology, Ministry of Agriculture and Rural Affairs of the People’s Republic of China, Beijing, China; ^4^Molbreeding Biotechnology Company, Shijiazhuang, China

**Keywords:** simple sequence repeat, target SSR-seq, cucumber, genetic diversity, DNA fingerprint

## Abstract

Simple sequence repeats (SSR) – also known as microsatellites – have been used extensively in genetic analysis, fine mapping, quantitative trait locus (QTL) mapping, as well as marker-assisted selection (MAS) breeding and other techniques. Despite a plethora of studies reporting that perfect SSRs with stable motifs and flanking sequences are more efficient for genetic research, the lack of a high throughput technology for SSR genotyping has limited their use as genetic targets in many crops. In this study, we developed a technology called Target SSR-seq that combined the multiplexed amplification of perfect SSRs with high throughput sequencing. This method can genotype plenty of SSR loci in hundreds of samples with highly accurate results, due to the substantial coverage afforded by high throughput sequencing. We also detected 844 perfect SSRs based on 182 resequencing datasets in cucumber, of which 91 SSRs were selected for Target SSR-seq. Finally, 122 SSRs, including 31 SSRs for varieties identification, were used to genotype 382 key cucumber varieties readily available in Chinese markets using our Target SSR-seq method. Libraries of PCR products were constructed and then sequenced on the Illumina HiSeq X Ten platform. Bioinformatics analysis revealed that 111 filtered SSRs were accurately genotyped with an average coverage of 1289× at an extremely low cost; furthermore, 398 alleles were observed in 382 cucumber cultivars. Genetic analysis identified four populations: northern China type, southern China type, European type, and Xishuangbanna type. Moreover, we acquired a set of 16 core SSRs for the identification of 382 cucumber varieties, of which 42 were isolated as backbone cucumber varieties. This study demonstrated that Target SSR-seq is a novel and efficient method for genetic research.

## Introduction

Simple sequence repeats (SSR) – otherwise known as microsatellites – exist ubiquitously throughout prokaryotic and eukaryotic genomes (Tóth et al., 2000). Based on their universal distribution and high density in a multitude of genomes, SSRs have been analyzed as second-generation molecular markers. Given their high rates of mutation, SSRs are widely used in genetic analysis, gene mapping, quantitative trait locus (QTL) mapping, and marker-assisted selection (MAS) breeding. SSRs in DNA coding regions are used as anchor markers for specific populations due to their homology among related species, while the large variations in SSRs found in non-coding regions provide adequate polymorphisms to distinguish related species. Hence, SSR markers have been specifically applied in a variety of identification procedures, allowing for the successful construction of a DNA fingerprinting database that includes the cultivars of a number of crops, such as maize, wheat, and watermelon (Zhang et al., 2012; Tian et al., 2015; Wang et al., 2015, 2017). However, many SSRs used in previous studies were often less polymorphic and failed to yield the expected PCR products. This limited the use and accuracy of SSR markers for genotyping in genetic research (Gao et al., 2012; Hu et al., 2014; Li et al., 2017).

Traditional gel electrophoresis cannot distinguish base differences or changes correctly in SSR amplicons, often causing false positive or false negative results in SSR detection, likely caused by sequence variations in the SSR motifs or their flanking sequences; these variations may affect the PCR process and hence, the resultant products. Recently, genome-wide analyses of SNPs, SVs, and transposon insertion polymorphisms (one of several transposable elements or TEs) were conducted based on large-scale resequencing studies in genetic variome research (Qi et al., 2013; Yang et al., 2017). However, few studies have focused on genome-wide SSRs, especially perfect SSRs, which exhibit stable motifs and conserved corresponding flanking sequences. To date, few studies have attempted to characterize genome-wide perfect SSRs. The few that do exist in the literature have focused on the SSR motifs themselves without looking further into their flanking sequences (Ding et al., 2017; Yasodha et al., 2018). Therefore, the identification of genome-wide perfect SSRs with stable motifs and corresponding flanking sequences that are highly conserved is critical in crops, such that amplification of the appropriate PCR products can be ensured in genetic research applications. It will remain impossible for the research community to achieve this goal without access to a high throughput technology for SSR genotyping.

A recent study established Ampli-seq as the first high throughput SSR genotyping method based on second-generation sequencing technology utilizing the Illumina MiSeq platform (Li et al., 2017). This study reported that the cost for the capture and detection of multiple SSRs in each rice line was $40 and $5, respectively; an average of 2427.75 SSRs was obtained out of a total of 3105 SSR targets in eight rice lines, with SSR coverage of 1855.38 and a genotyping success rate of 78.19% (Li et al., 2017). However, the rapid development of high throughput sequencing technology has yielded several novel and more economical sequencing platforms, such as the Illumina HiSeq X Ten (X Ten) and the NovaSeq (Meynert et al., 2014; Costello et al., 2018). These instruments provide opportunities to develop novel high throughput SSR genotyping technologies when combined with genome-wide perfect SSR discovery at a lower cost and with higher success rates than currently available methods.

In this study, we developed a novel method called Target SSR-seq, which combined the high throughput sequencing system X Ten platform with genome-wide perfect SSRs that harbored stable motifs and flanking sequences derived from 182 resequencing datasets of a core collection of cucumber lines. This method enables the genotyping of hundreds of targeted SSR loci in a large number of samples with high coverage, simultaneously in a single Illumina HiSeq lane (Yang et al., 2016). By adding sequencing adapters and dual barcode tags (Campbell et al., 2015), the SSR genotypes were determined directly from the deep sequencing (∼1000×) of PCR products. The present study constructed the DNA fingerprints of 382 cucumber varieties with 89 genome-wide perfect SSRs and 22 well-known SSRs for varieties identification using the Target SSR-seq technology. The analysis required 72 h for high throughput genotyping at a cost of $7 for each variety, demonstrating the high utility of this new approach. This study developed a core set of perfect SSRs in cucumber, including backbone varieties, which demonstrated their breeding history in China.

## Materials and Methods

### Plant Materials and DNA Extraction

A total of 382 commercial cucumber varieties were utilized in this study (Supplementary Table S1), including 115 varieties from the seed department of the Chinese government, 146 varieties from breeders, 91 commercial hybrid varieties from seed markets, and 31 varieties cultivated in Xishuangbanna. First true leaves from 30 independent individuals, which was required based on the National Varieties Identification Standard, were collected and mixed to extract DNA following a CTAB-based method in this study (Stewart and Via, 1993).

### Discovery of Genome-Wide Perfect SSRs in Cucumber

First, the cucumber reference genome 9930 V2 was analyzed to uncover genome-wide SSRs using GMATA software with the following parameters: motif repeated at least three times, motif length at least 3 bp, and repeat length up to 100 bp (Huang et al., 2009; Wang and Le, 2016). In order to select the suitable SSR loci for Target SSR-seq, we extracted SSRs with 2 bp motif repeats at least six times, 3 bp motif repeats at least five times, 4 bp motif repeats at least four times, 5 bp motif repeats at least three times, and 6 bp motif repeats at least two times. Moreover, 15-bp flanking sequences of SSR loci on the reference genome were mapped to reference genome using BWA, and SSRs with unique matches were retained. Second, a collection of resequencing data from 182 genetically diverse cucumber accessions, including the 115 published lines (SRA056480 in NCBI, Qi et al., 2013) as well as the 67 unpublished resequencing data (Supplementary Table S2), was used to discover genome-wide perfect SSRs. The perfect SSRs were constrained using the following criteria: (i) SSR motif length less than 50 bp; (ii) no INDELs, poly regions, and SSR loci in the 150 bp flanking sequence; (iii) read frequency of the major SSR allele in one accession greater than 0.7 to reduce the noise when BWA allowed mismatch; (iv) PIC value greater than 0.3 to ensure the SSR polymorphism in varieties. (v) Even distribution in chromosomes. Finally, a multiplexed PCR panel of the selected perfect SSRs was designed by Molbreeding Biotechnology Company (Shijiazhuang, China).

In addition, 58 well-known SSR loci used to distinguish cucumber varieties in China (NY/T 2474-2013) (Lv et al., 2012) were analyzed based on the criteria for multiplexed PCR, 31 of which were retained to compare genotyping efficiency to that in the perfect SSRs.

### Target SSR-Seq Library Construction

**FIGURE 1:**
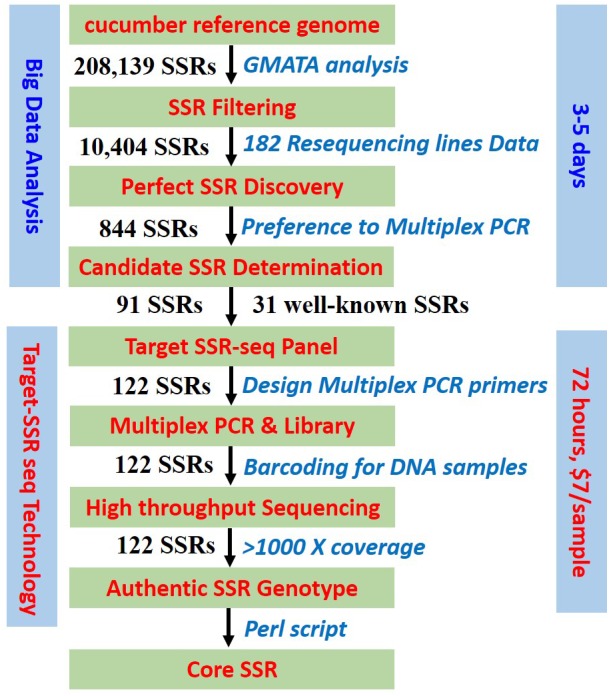
Target SSR-seq pipeline. Schematic workflow of perfect SSR selection, Multiplexed PCR design, high-throughput sequencing, and authentic SSR genotype.

The Target SSR-seq library construction consisted of two rounds of PCR (Figure 1): the first round amplified and captured the target SSRs in plant DNA samples using a multiplexed PCR panel; the second round added a unique barcode to the capture product for each DNA sample. First, the multiplexed PCR was conducted in 30 μL reactions including 50 ng DNA template, 10 μL of 3 M enzymes, and 8 μL of the multiplexed SSR-capture panel mix (Molbreeding Biotechnology Company, Shijiazhuang, China). The PCR conditions were as follows: 95°C for 5 min then 17 cycles of 95°C for 30 s and 60°C for 4 min, and extension at 72°C for 4 min. The PCR products were purified by magnetic bead suspension and 80% alcohol. Then the second PCR was performed in 30 μL reactions consisting of 11 μL of purified PCR product from the previous round, 10 μL of 3 M *Taq* enzyme, 18 μL pure water, and 1 μL of primers with the following sequences: forward 5′-AATGATACGGCGACCA-CCGAGATCTACACTCTTTCCCTACACGACGCTCTTCCG-3′ and reverse 5′-CAAGCAGAAGACGGCATACGAGATXXXXXXXXGTGACTGGAGTTCCTTGGCACCCGAGA-3′ (barcodes are indicated by underlined sequences). The PCR conditions were as follows: 95°C for 3 min then 7 cycles of 95°C for 15 s, 58°C for 15 s, and 72°C for 30 s, and extension at 72°C for 4 min. The second round PCR products were purified with 100 μL 80% alcohol and 23 μL Tris–HCl buffer (10 mM, pH 8.0–8.5). Thereafter, the Target SSR-seq library was ready to sequence on the X Ten platform (Molbreeding Biotechnology Company). To verify the repeatability of Target SSR-seq, DNA of cucumber 9930 and pure water were set up as positive and negative controls in PCR amplification, respectively.

### SSR Genotyping Analysis of Target SSR-Seq

The raw data reported in this study have been deposited in the Genome Sequence Archive^1^ under accession numbers CRA001490. The raw Target SSR-seq data were de-multiplexed to determine the exact genotypes for each variety using the Illumina bcl2fastq pipeline (Illumina, San Diego, CA, United States). Adaptor and low-quality sequences were filtered out from raw reads using Trimmomatic with parameters as “SLIDINGWINDOW: 4:20 LEADING:3 TRAILING:3 MINLEN:40” (Bolger et al., 2014). The reads of each variety were mapped to the cucumber reference genome (9930 V2)^2^ using BWA with default parameters and 15-bp flanking sequences of SSR loci on reference genome were isolated to determine the perfect SSR genotype using MISA software^3^ (Li and Durbin, 2009). Based on the high-throughput sequencing results, the SSR alleles with the maximum numbers of reads and the second maximum numbers of reads were treated as the major and minor allele for each target SSR loci. When the read frequency of the major allele was more than 0.7, this locus was described as homozygous. When the read frequencies of the major and minor alleles were both more than 0.35, this locus was treated as heterozygous.

### Genetic Information Statistics for Target SSRs

Genetic information statistics including SSR allele number per locus, observed heterozygosity (*H_o_*), genetic diversity, polymorphic information content (PIC) value (Botstein et al., 1980), and inbreeding coefficient (F, Wright, 1965) were calculated using a Perl script with the following equation:

$$\begin{array}{c} PIC=1-\sum_{i=1}^{l}P_{i}^{2}-\sum_{i=1}^{l-1}\sum_{j=i+1}^{l}2P_{i}^{2}P_{j}^{2}\\ Genetic\;diversity=1-\sum_{i=1}^{l}P_{i}^{2}\\ F=1-\frac{H_{o}}{H_{e}} \end{array}$$

where *l* is the allele locus and *P_i_* and *P_j_* represent the population frequency of the *i*th and *j*th allele.

### Genetic Structure Analysis in Cucumber Varieties

Population structure was inferred by a model-based program STRUCTURE V2.3 with the following parameters: 100,000 burn-in length, 10,000 iterations, admixture model (Pritchard et al., 2000; Falush et al., 2003). The optimal number of ancestors (K) was determined using the ΔK method with K ranging from 1 to 10. The population of individuals was defined by the proportional membership. Furthermore, a “hierarchical STRUCTURE analysis” was applied to suspect the potential subpopulation structure (Vähä et al., 2007; Emanuelli et al., 2013). A hierarchical clustering on principal components (HCPC) analysis was performed to validate the results defined by STRUCTURE, with HCPC function in the FactoMineR R package (Lê et al., 2008; Husson et al., 2014). The variance between clusters, variance gain, and variance ratio were calculated with the cluster number Q ranging from 1 to 10. The optimal cluster was determined by the minimized variance ratio. In addition, a principal co-ordinate analysis (PCoA) and an unrooted Neighbor-joining tree with Nei’s standard genetic distance were performed using the ape and poppr packages in R software (Nei, 1978; Kamvar et al., 2014).

### Population Differentiation Analysis in Cucumber Varieties

To measure genetic differentiation between populations, we performed an analysis of molecular variance (AMOVA); the pairwise *Fst.* AMOVA was performed in the poppr R package (Kamvar et al., 2014) and the pairwise *Fst* was performed with the hierfstat R package.

### Core SSRs Set Exploration for Varieties Identification

To select a core SSR set for variety identification, we developed a new Perl method to choose the best discernibility group based on the principle of minimum numbers of SSRs representing the maximum genetic diversity. Discernibility by pairwise comparison of all samples was as the first filter condition, and the dataset with the same discernibility were then selected with higher PIC. The highest discernible SSR loci were chosen as an initial core dataset and each SSR were subsequently added to the initial core dataset to form a new dataset. The second SSR were chosen from the new dataset with highest discernibility and were added to the core dataset. The following selection were the same as the second SSR until the discernibility reached the maximum. Finally, a best-discernibility group of SSRs was obtained as the core SSR set, and the saturation curves of its discernibility were plotted by pairwise comparison of varieties genotypes.

### Core Varieties Analysis in Chinese Cucumber Markets

According to the international standards for identifying crop varieties (International Union for the Protection of New Varieties of Plants [UPOV], 2011), we set up a pairwise comparison matrix by calculating the numbers of differential SSR genotypes between each variety and the remaining ones; the missing genotype was treated as null. Fewer differential SSR genotypes indicated closer kinship with others. The top 10% of varieties with close kinship were considered core varieties in each group.

## Results

### The Novel Target SSR-Seq Pipeline

In this study, we developed a novel approach for SSR genotyping using a target sequencing technology called Target SSR-seq, which can be applied in genetic research, DNA fingerprinting, variety identification, and molecular breeding (Figure 1). This study tested the Target SSR-seq pipeline in cucumber, the genome of which is well assembled (Huang et al., 2009; Li et al., 2011). First, we selected the candidate SSR loci to be genotyped. Second, we designed a multiplexed PCR procedure to capture target SSR regions in a plant genome. Then the Target SSR-seq library was sequenced on the high-throughput sequencing platform X Ten; each SSR region was sequenced for at least 1000× coverage. To assay the repeatability of Target SSR-seq, positive and negative controls of PCR amplification were set. The cucumber 9930 was set up as a positive control, and the pure water was used as a negative control in the Target SSR-seq experiment. The amplification and sequencing result showed that the genotype of 91 perfect SSRs was the same to that in the cucumber reference genome (9930 V2). While as the negative control showed no PCR bands after screening in agarose electrophoresis. The positive and negative controls proved that Target SSR-seq could obtain reliable genotyping result.

Compared with existing methods for SSR detection, Target SSR-seq combined multiplexed PCR with target deep sequencing and was immediately capable of highly accurate SSR genotyping (Supplementary Table S3). This new technology successfully genotyped hundreds of target SSR loci in numerous samples within 72 h at a cost of $7 for each variety, which was more efficient and cost-effective than the previously reported Amp-Seq SSR technology (Supplementary Table S3).

### Discovery of Genome-Wide Perfect SSRs for Target SSR-Seq

We acquired 208 139 SSRs in the cucumber reference 9930 V2 genome, of which 10 404 SSRs were suitable for multiplexed PCR capture. Based on the resequencing data for 182 cucumber varieties, 1700 SSRs exhibited polymorphisms. Furthermore, 844 perfect SSRs were obtained with read frequency of the major alleles greater than 0.7 and stable flanking sequences. In this study, 91 evenly distributed perfect SSRs in the cucumber genome were randomly selected to test in Target SSR-seq.

In addition, the current Target SSR-seq panel included 31 SSR loci that are often tested in genetic research on cucumbers, which were used to compare the genotyping efficiency with that of the genome-wide perfect SSRs. Finally, a total of 122 target SSRs were successfully designed in the next multiplexed PCR procedure.

### Genotyping Analysis in Target SSR-Seq

**FIGURE 2:**
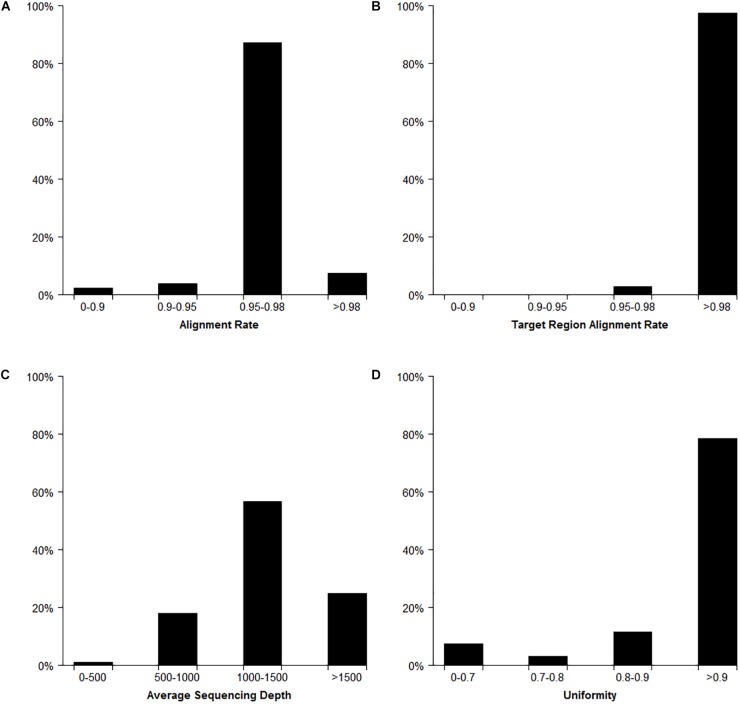
Target SSR-seq genotyping result analysis. The distribution of reads alignment **(A)**, target region alignment **(B)**, average read depths **(C)**, and uniformity index **(D)** for 382 cucumber varieties.

In total, the Target SSR-seq obtained 230 million reads and 34 billion bases in 382 cucumber varieties (Figure 2). In the 122 target SSR loci, six SSRs from the 31 compared SSRs failed to be genotyped due to low motif capture (80.6% success rate), while the 91 tested perfect SSRs were successfully genotyped (100%). The average coverage of the 116 retained SSRs in each sample was 1289× (Figure 2, 3A). Among the 382 varieties, 375 varieties (98%) showed more than 90% alignment rate to the 9930 V2 reference genome (Figure 2A). Out of these aligned reads, 372 varieties (97.4%) exhibited an alignment rate to the target SSR motif over 98%, and the alignment rate in all 382 varieties was above 95% (Figure 2B). The average read depth per SSR capture in 311 varieties (81.5%) was more than 1000× (Figure 2C). Furthermore, we analyzed the Target SSR-seq uniformity index, in order to calculate the proportion of the coverage above 10% of mean depth value for each variety (Nishio et al., 2015). The average uniformity index in this study was 89.5% (Figure 2D), indicating a higher uniformity index and more accurate results. Moreover, two SSRs from the 25 retained for comparison harbored no polymorphisms in 382 varieties, and one SSR exhibited a high miss rate (>20%), probably due to an unstable flanking sequence. Two of the 91 perfect SSRs were observed as monomorphisms in 382 varieties. Finally, we obtained 111 polymorphic SSRs for genotyping 382 cucumber varieties from Chinese markets.

### Genetic Diversity of Cucumber Varieties in China

**FIGURE 3:**
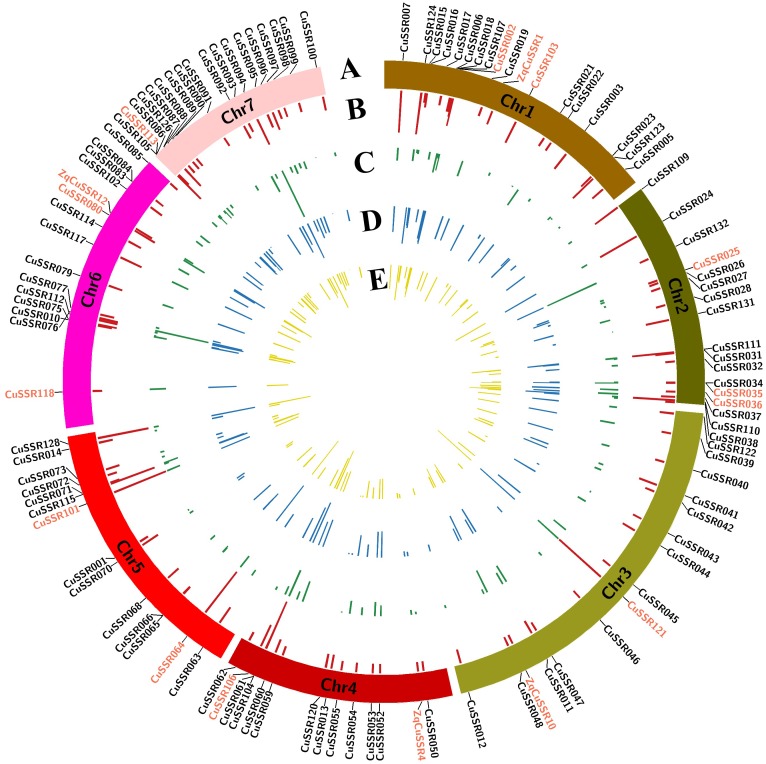
Genetic characterization of 111 SSRs in 382 cucumber varieties. **(A)** Distribution of 111 SSR loci in seven cucumber chromosomes. 16 core SSR set is labeled in red. **(B)** Allele numbers per SSR locus. **(C)** Observed heterozygosity. **(D)** Genetic diversity. **(E)** PIC value.

Target SSR-seq captured 398 alleles of 111 target SSR loci in 382 varieties and the allele number per SSR locus varied from 2 to 12 with an average of 3.6 (Figure 3B). Trinucleotides and dinucleotides were the first two motif types, accounting for 37.4 and 28.8% in 398 alleles, respectively (Supplementary Figure S1A). The SSR motif repeats ranged from 2 to 23, and 127 alleles (32%) contained two repeat units (Supplementary Figure S1B). There were 239 alleles (60%) with minor allele frequency (MAF) above 5% that were regarded as common alleles, while only 20.3% were found in a previous study (Lv et al., 2012). In the 159 rare alleles, 28 (17.5%) were specifically observed in Xishuangbanna varieties. We found the observed heterozygosity *Ho* varied from 0 to 0.95 with a mean of 0.17, and seven SSRs exhibited higher *Ho* (>0.4) (Figure 3C). The low *Ho* indicated a narrow genetic background in 382 Chinese cucumber varieties. Furthermore, the genetic diversity estimated by expected heterozygosity varied from 0.003 to 0.809 (mean = 0.367, Figure 3D), while the PIC value ranged from 0.003 to 0.782 (mean = 0.310, Figure 3E). Interestingly, the inbreeding coefficient of four perfect SSR loci was negative, indicating that these loci had excess heterozygosity. Overall, the 111 target SSR loci showed various alleles and high polymorphism rates, which were proven to be suitable for varieties identification.

### Genetic Structure of Cucumber Varieties in China

**FIGURE 4:**
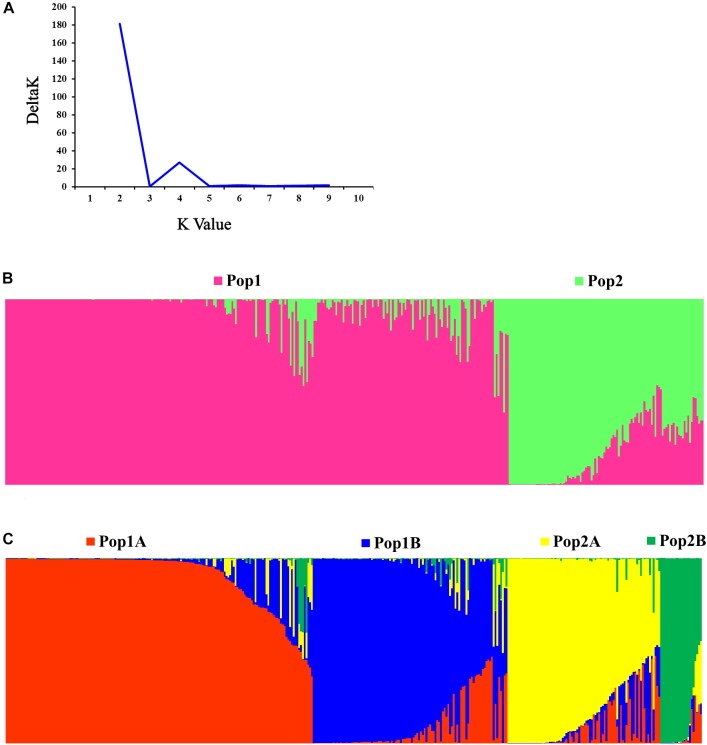
Population structure of 382 cucumber varieties. **(A)** Delta K plots derived from Target SSR-seq result. **(B)** Two populations were observed in 382 varieties, Pop1 is colored in pink and Pop2 is colored in green. **(C)** Four subpopulations were classified and Pop1A, Pop1B, Pop2A, and Pop2B are colored with red, blue, yellow, and green, respectively.

The STRUCTURE and Evanno’s correction results indicated that 382 cucumber varieties were divided into two main populations (Pop1 and Pop2), based on the optimal number of K = 2 (Figure 4A,B). In general, 276 cucumber varieties (72.1%) were assigned to Pop1 and the remaining 107 varieties were assigned to Pop2. To detect the subpopulation structure, a hierarchical STRUCTURE analysis was performed. The Pop1 was divided into Pop1A and Pop1B, while the Pop2 was composed of Pop2A and Pop2B (Figure 4C). A total of 99% division defined by “hierarchical STRUCTURE analysis” was the same as those retrieved from the first round STRUCTURE analysis when K = 4, which agreed with the plateau criterion proposed by Pritchard et al. (2010). According to its geographic origin, Pop1A belonged to northern China cucumber, Pop1B indicated the southern China cucumber, while Pop2A represented cucumber derived from Europe and Pop2B inferred the unique Xishuangbanna cucumber.

**FIGURE 5:**
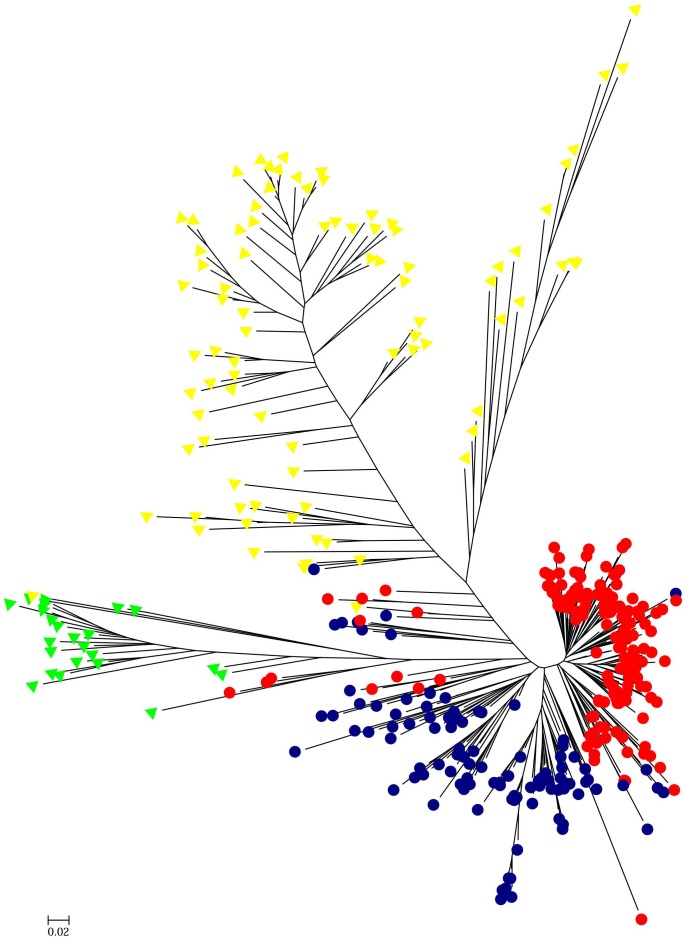
Unrooted neighbor-joining tree of 382 cucumber varieties. The Pop1A, Pop1B, Pop2A, and Pop2B subgroups are colored the same as in Figure 4.

**FIGURE 6:**
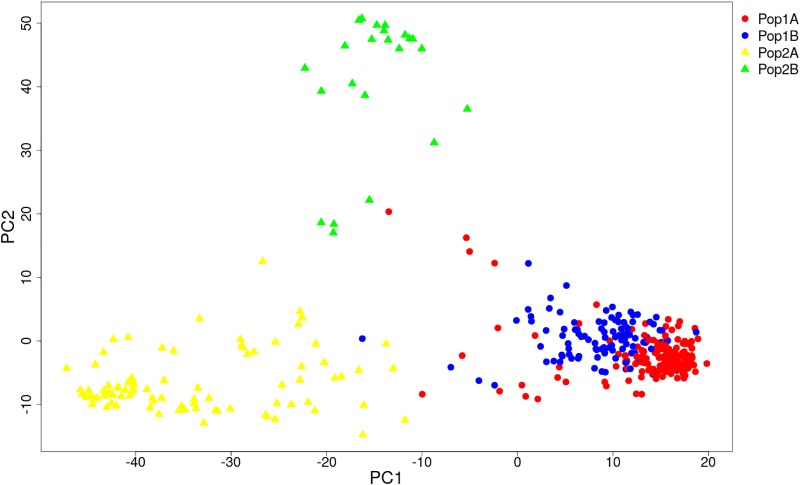
Principal co-ordinates analysis (PCoA) of 382 cucumber varieties. The Pop1 and Pop2 are labeled with circle and triangle. The four subpopulations are colored the same as in Figure 4.

HCPC analysis was used to validate the results from STRUCTURE. The variance between cluster and the variance gain were significantly decreased when the cluster number increased (Supplementary Figure S2). The recommended two clusters inferred by the minimum variance ratio was consistent with analysis on STRUCTURE. However, the variance gain increased slowly with cluster numbers beyond four (Supplementary Figure S2), indicating that four distinct sub-clusters existed. Furthermore, a hierarchical clustering tree also demonstrated two clusters and four sub-clusters (Supplementary Figures S3, S4). Moreover, the unrooted Neighbor-joining (NJ) tree and Principal co-ordinates analysis (PCoA) indicated a clear distinction in two populations and four subpopulations, despite the fact that Pop1B was close to Pop1A (Figure 5, 6). Figure 5 and Supplementary Figure S3 also showed that Pop2A were divided into two branches, one was typical European fruit types, and the other one was European fruit types which interbreed with southern China cucumber.

### Population Differentiation of Cucumber Varieties in China

AMOVA analysis of 111 SSR genotypes in 382 varieties indicated that the maximum variation of 29.2% resulted from differences within samples, while the minimum variation of 17.6% was accounted for between subpopulations within populations (Table 1). The *Fst* result demonstrated that population differentiation between Pop1 and Pop2 is moderate (*Fst* = 0.35), which was similar to previous research in cucumber germplasms, ranging from 0.30 to 0.33 based on 23 SSRs (Lv et al., 2012). The pairwise *Fst* between four subpopulations ranged from 0.14 to 0.47 (Table 2). Among them, the *Fst* between Pop1A and Pop1B showed a low level of differentiation (*Fst* = 0.14). The distinct differentiation was observed in other pairwise *Fst* analysis.

**Table 1:** Analysis of molecular variance (AMOVA) among populations and within populations.

Source of variation	*df*	Sum of squares	Variance components	Percentage of variation
Between populations	1	2927.7	6.7	26.7
Between subpopulations Within populations	2	1502.0	4.4	17.6
Between samples Within subpopulations	378	7763.4	6.6	26.5
Within samples	382	2778.5	7.3	29.2
Total	763	14971.6	25.0	100

**Table 2:** Pairwise differentiation *Fst* among four subpopulations.

Population	Pop1B	Pop2A	Pop2B
Pop1A	0.1414	0.4623	0.4738
Pop1B	–	0.3973	0.4181
Pop2A	–	–	0.4135

### Core SSR Set in Cucumber Varieties Identification

**FIGURE 7:**
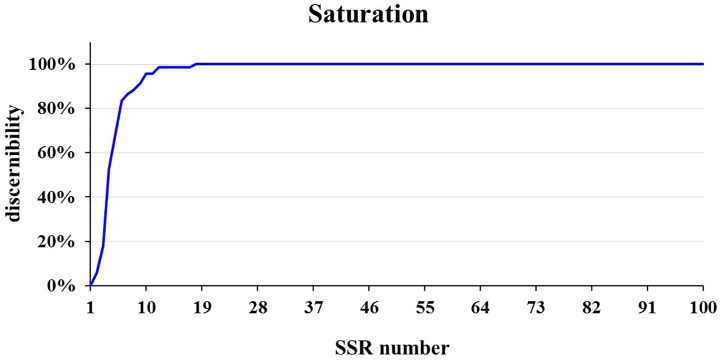
The saturation curve of 111 SSRs identifying in 382 cucumber varieties. A total of 16 SSRs identified 99% cucumber varieties.

The core SSR set was used to analyze the genetic diversity and variety identity in crops (Lv et al., 2012; Zhang et al., 2016). This study found that a set of 16 core SSRs could distinguish 99% of 382 commercial cucumber varieties (Figure 2A, 7 and Supplementary Table S4) and the similar varieties (1%) could be distinguished with two SSRs. Structure analysis based on 16 SSRs classified the 382 varieties into two populations (Supplementary Figure S5). The PCoA analysis significantly distinguished the two populations with PC1 explained by 18.6% and PC2 explained by 9.4%, respectively (Supplementary Figure S6). The AMOVA analysis showed that the variations were evenly distributed in populations, samples within populations, and within samples (Supplementary Table S5). The pairwise *Fst* between Pop1 and Pop2 was 0.25. Hence, this set of 16 core SSRs was sufficient in representing the genetic diversity and identifying cucumber varieties in Chinese markets.

### Genetic Similarity and Core Varieties Analysis

The genetic background of Chinese cultivated cucumber was considerably narrow, given that breeders follow similar breeding goals, resulting in many varieties with close genetic relationships. In this study, we built a genetic similarity matrix in four subgroups by counting the number of differential SSR genotypes between each DNA sample (Supplementary Figure S6). High genetic similarity was observed within cucumbers belonging to northern China type, suggesting a long breeding history and extensive gene exchange in this group (Supplementary Figure S7A), while the European cucumber type exhibited high genetic diversity, according to its recent introduction into China (Supplementary Figure S7C). Among 382 cucumber varieties, “Jinyou1hao” had the minimum number of differential SSR genotypes with others, while a European variety “Virginia” had the maximum differential SSR genotypes. We selected the top 10% of varieties with minimum differential SSR genotypes as core varieties within each subgroup. Finally, 42 varieties were identified and considered to be core or backbone varieties of 382 cucumber varieties, which was in accordance with breeders’ views (Supplementary Table S6).

## Discussion

### High Accuracy and Efficiency of Target SSR-Seq

Simple sequence repeats (SSRs), also known as short tandem repeats (STRs) or microsatellites, exist extensively throughout eukaryotic genomes and are therefore used widely in genetic background selection and MAS breeding, as well as in map-based cloning, QTL mapping, seed identification and purification (Wang et al., 2017). However, few studies have focused on the accuracy and authenticity of SSR genotypes. Due to the high number of variations existing in SSR motifs and flanking regions, the available methods for SSR genotyping often generate false positive or false negative results (Li et al., 2017). Therefore, the research community needs development of novel methods for perfect SSR discovery and genotyping that require less time and cost less, while delivering high accuracy and efficiency. In this study, Target SSR-seq genotyped hundreds of perfect SSRs using a high-throughput resequencing method that yielded accurate results due to coverage as high as 1289× (Figure 2C). Moreover, the positive control result showed that the Target SSR-seq of cucumber 9930 obtained the same genotyping results with that in reference genome sequence (9930 V2). And the negative control result showed no PCR amplification. This proved that the Target SSR-seq could gain preferable repeatability. Compared to traditional SSR genotyping methods, the efficiency of Target SSR-seq is hundreds of times higher, acquiring dozens to thousands of datapoints in 72 h at a cost less than $7 for each sample. Compared to the recently reported Amp-Seq SSR method (Li et al., 2017), our study gained a genotyping success rate of 100% based on perfect SSRs while 78% was obtained with Amp-Seq SSR; Target SSR-seq also requires less time and fewer consumable materials by utilizing a high-throughput sequencing platform (Figure 1). In addition, the 100% success rate of 91 perfect SSRs was more than the 80.6% of 31 compared SSRs, commonly used in previous studies (Lv et al., 2012). Therefore, Target SSR-seq succeeds in providing high-throughput SSR genotyping with high accuracy and efficiency for genetic research.

### Powerful Application in Varieties Identification of Target SSR-Seq

With the development of domestic and international seed trade, the commercial quality of seed based on authenticity and purity is becoming more important for both seed producers and farmers (Gao et al., 2012). The traditional way to measure seed authenticity and purity relies on field investigation, which is time-consuming and labor-intensive and unsuited for the fast-paced inspection demands of today (Tian et al., 2015). Recently, UPOV (the International Union for Protection of New Varieties of Plants) proposed SSR markers for variety identification and DNA fingerprinting data base construction (International Union for the Protection of New Varieties of Plants [UPOV], 2011). To date, DNA fingerprinting database using SSR markers was successfully built in cultivars such as rice, maize, wheat, watermelon, cucumber, and melon. However, the sequence variations of motif and flanking regions in these SSRs were not clearly known, causing a certain amount of SSRs to yield poor results when screened in diverse genetic accessions. Amp-Seq SSR as a new method was able to genotype more than thousands of SSRs at once using high throughput-sequencing technology and was successfully applied in rice research (Li et al., 2017). Moreover, it is convenient to use fewer numbers of SSR markers rather than thousands of markers in identifying varieties, especially in vegetable crops due to small genomes and limited numbers of varieties in markets. Thus, this study calculated a core set of 16 perfect SSRs to identify varieties and set up DNA fingerprints successfully. Consequently, Target SSR-seq combined with perfect SSRs is a powerful method for genetic analysis and varieties identification.

### Genetic Diversity Analysis in Chinese Cucumber Varieties

It was well known that China has a long history in the cultivation of cucumbers since the Han dynasty, when it has been reported that cucumber was first introduced into China through the Silk Route (Lv et al., 2012). Over several thousand years of human selection and improvement, Chinese cucumbers have gained special features (Qi et al., 2013), especially in fruit length. Over the last 30 years, many modern European varieties and resources were again introduced to China, improving the traditional Chinese cucumber varieties. To date, China is the world’s top producer and consumer of cucumbers, with over 1.16 million hectares in cultivated acreage and about 61.9 million tons of production in 2016^4^. However, the genetic background has remained unclear, as well as the diversity of cucumber varieties in current Chinese markets. This study created a novel method called Target SSR-seq, which successfully genotyped 111 genome-wide SSRs in 382 cucumber varieties in Chinese markets. The results showed four subpopulations were found: northern China type, southern China type, European type, and Xishuangbanna type (Figure 4C), which was consistent with the geographic distributions (Lv et al., 2012). However, including material from India is likely to change these patterns. In addition, we identified 42 core cucumber varieties by counting the number of differential SSR genotypes of each variety compared to other ones (Supplementary Figure S7), which was inconsistent with the definition of core resources collection. The core varieties generally harbored more common alleles within groups. Accordingly, the Jingyouyihao variety had high genetic similarity with other varieties, and several Europe varieties had high genetic diversity compared with other groups. This was in accordance with cucumber breeding history in past decades.

### Potential Applications of Target SSR-Seq

In view of its high accuracy and efficiency, Target SSR-seq associate with genome-wide perfect SSRs has great potential application not only in varieties identification, but also in many other research fields (Zhang et al., 2012; Weng et al., 2015; Li et al., 2017), such as genetic background selection, gene mapping procedures, QTL mapping, and molecular breeding. Furthermore, the Target SSR-seq technology provides a great potential opportunity to utilize well-studied SSRs explored by the global research community, in order to set up a novel molecular design breeding panel. To date, there were dozens of published functional SSRs in cucumber, like powdery mildew resistance (He et al., 2013), early flowering (Lu et al., 2014), perfect flower (Tan et al., 2015), female flower time (Bo et al., 2015), fruit peduncle length (Song et al., 2016); parthenocarpy (Lietzow et al., 2016; Wu et al., 2016), downy mildew resistance (Wang et al., 2016), fruit length (Pan et al., 2017), and waterlogging (Xu et al., 2017). Combining these functional SSRs with target SSR-seq technology, this technology would be applied in a breeding system to greatly raise breeding efficiency and decrease pyramiding breeding period. In conclusion, Target SSR-seq can be widely used in many research fields.

## Author Contributions

CW designed the research. JY did the bioinformatics analysis. CW, RH, FZ, AM, and HT prepared the research. JianZ, JL, BD, and HL performed the research. JiananZ designed the multiple PCR. YJ, JianZ, and RH analyzed the data and wrote the manuscript. All authors read and approved the final manuscript.

## Conflict of Interest Statement

The authors declare that the research was conducted in the absence of any commercial or financial relationships that could be construed as a potential conflict of interest.

## Footnotes

1 http://bigd.big.ac.cn/gsa

2 http://cucurbitgenomics.org/

3 http://pgrc.ipk-gatersleben.de/misa/

4 http://www.fao.org/

## Supplementary Material

FIGURE S1 The distribution of motif repeats **(A)** and motif length **(B)** in 398 alleles for 382 cucumber varieties.

FIGURE S2 The variance in HCPC analysis. Variance in blue bars (left ordinate), variance gain (left ordinate) in orange bars and variance ratio in green bars (right ordinate) changed with cluster number.

FIGURE S3 Hierarchical tree produced by HCPC. Four branches (Pop1A, Pop1B, Pop2A, and Pop2B) were obtained and colored with red, blue, yellow, and green, respectively.

FIGURE S4 Principal component analysis of 382 cucumber varieties by HCPC. Pop1A, Pop1B, Pop2A, and Pop2B are labeled in red, blue, yellow, and green blocks, respectively.

FIGURE S5 Population structure analysis with 16 core SSRs set in identifying cucumber varieties. **(A)** Delta K plots derived from 16 core SSRs set in 382 varieties. **(B)** Two observed populations were consistent with results from 111 SSRs.

FIGURE S6 PCoA analysis with 16 core SSRs set. Pop1 and Pop2 are labeled in pink and green, respectively.

FIGURE S7 Heatmap of pairwise comparison matrix derive from differential SSR genotypes in Pop1A **(A)**, Pop1B **(B)**, Pop2A **(C)**, and Pop2B **(D)**. Red to Blue indicated the increasing differential SSR genotypes.

TABLE S1 Information of 382 cucumber varieties in Chinese market.

TABLE S2 Information of 67 unpublished resequencing data used in this study.

TABLE S3 Comparisons of Target SSR-seq in SSR genotyping with current methods.

TABLE S4 The genetic characteristic information of 16 core SSRs set in cucumber variety identification.

TABLE S5 Analysis of molecular variance (AMOVA) among populations based on 16 core SSRs set.

TABLE S6 Core cucumber varieties in Chinese market.
