# Supplementary figures and images for: Target SSR-Seq: A Novel SSR Genotyping Technology Associate With Perfect SSRs in Genetic Analysis of Cucumber Varieties
Source: Front Plant Sci. 2019 Apr 24;10:531. doi: 10.3389/fpls.2019.00531 (PMC6492046; doi:10.3389/fpls.2019.00531)

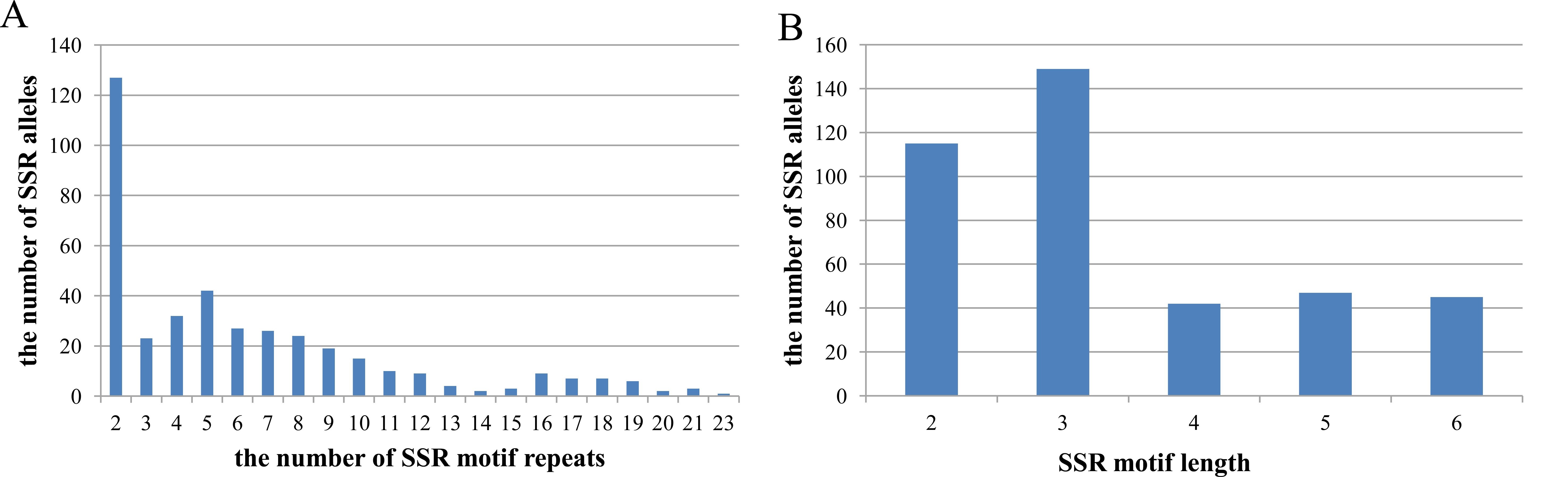

Supplement: FIGURE S1 — The distribution of motif repeats (A) and motif length (B) in 398 alleles for 382 cucumber varieties. [file Image_1.JPEG]

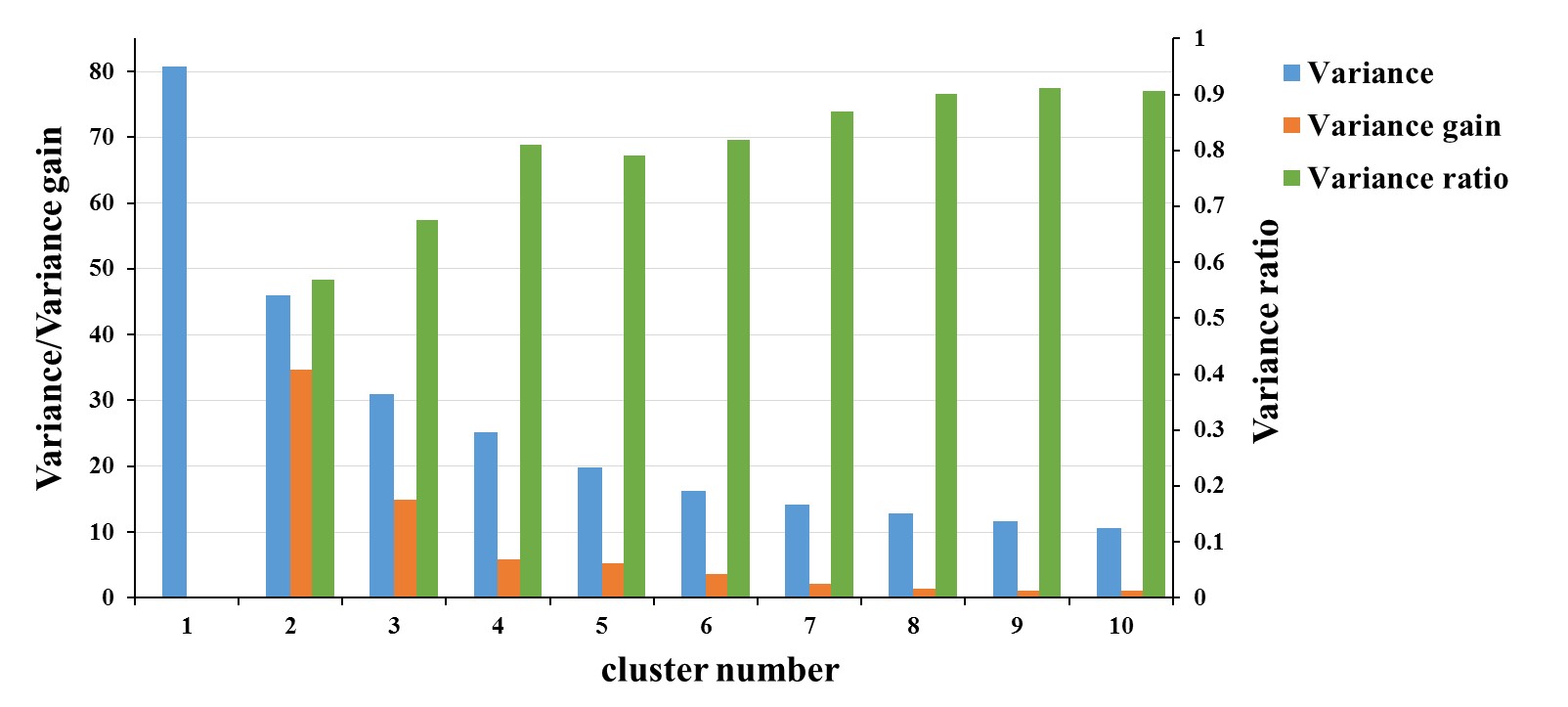

Supplement: FIGURE S2 — The variance in HCPC analysis. Variance in blue bars (left ordinate), variance gain (left ordinate) in orange bars and variance ratio in green bars (right ordinate) changed with cluster number. [file Image_2.JPEG]

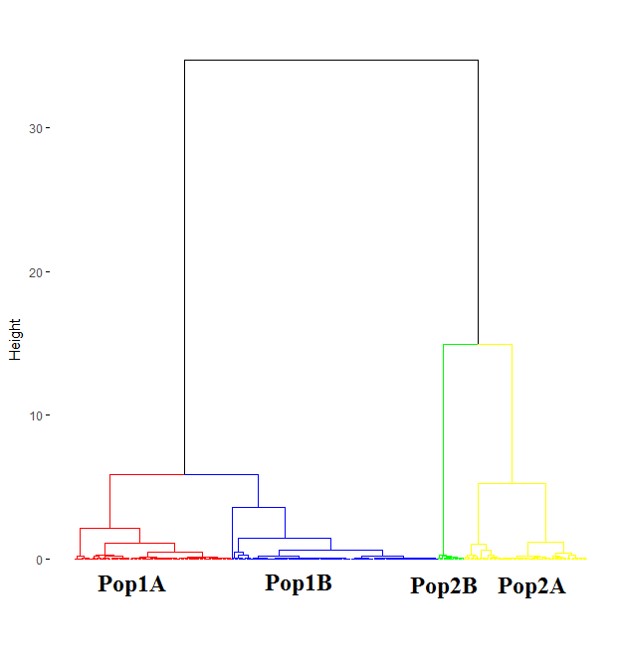

Supplement: FIGURE S3 — Hierarchical tree produced by HCPC. Four branches (Pop1A, Pop1B, Pop2A, and Pop2B) were obtained and colored with red, blue, yellow, and green, respectively. [file Image_3.JPEG]

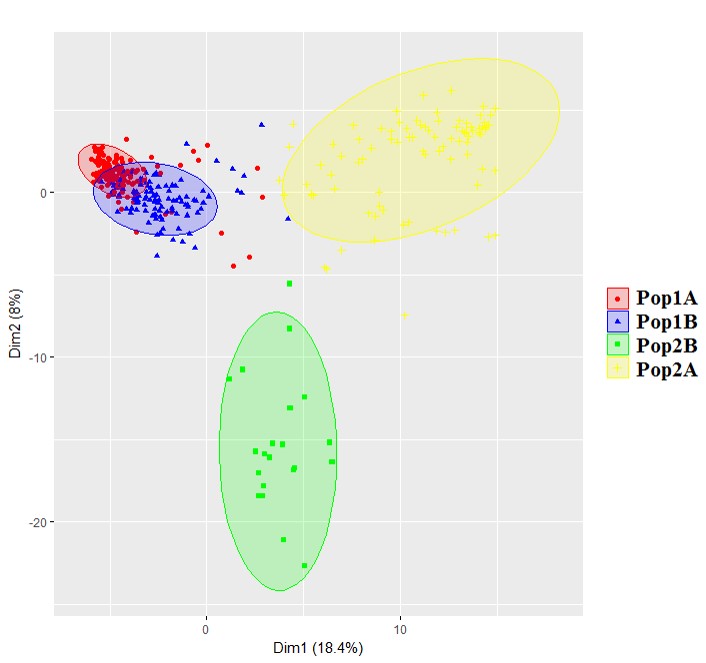

Supplement: FIGURE S4 — Principal component analysis of 382 cucumber varieties by HCPC. Pop1A, Pop1B, Pop2A, and Pop2B are labeled in red, blue, yellow, and green blocks, respectively. [file Image_4.JPEG]

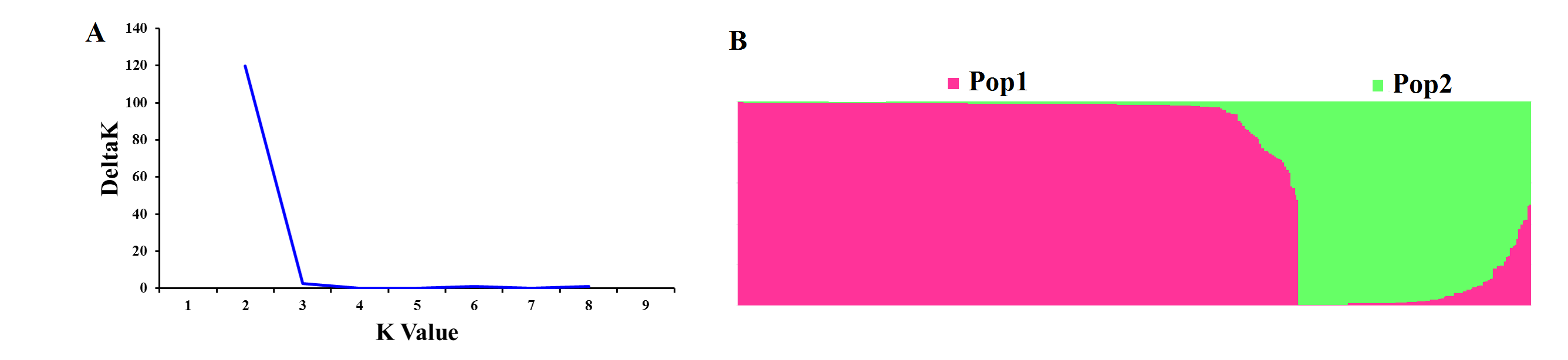

Supplement: FIGURE S5 — Population structure analysis with 16 core SSRs set in identifying cucumber varieties. (A) Delta K plots derived from 16 core SSRs set in 382 varieties. (B) Two observed populations were consistent with results from 111 SSRs. [file Image_5.JPEG]

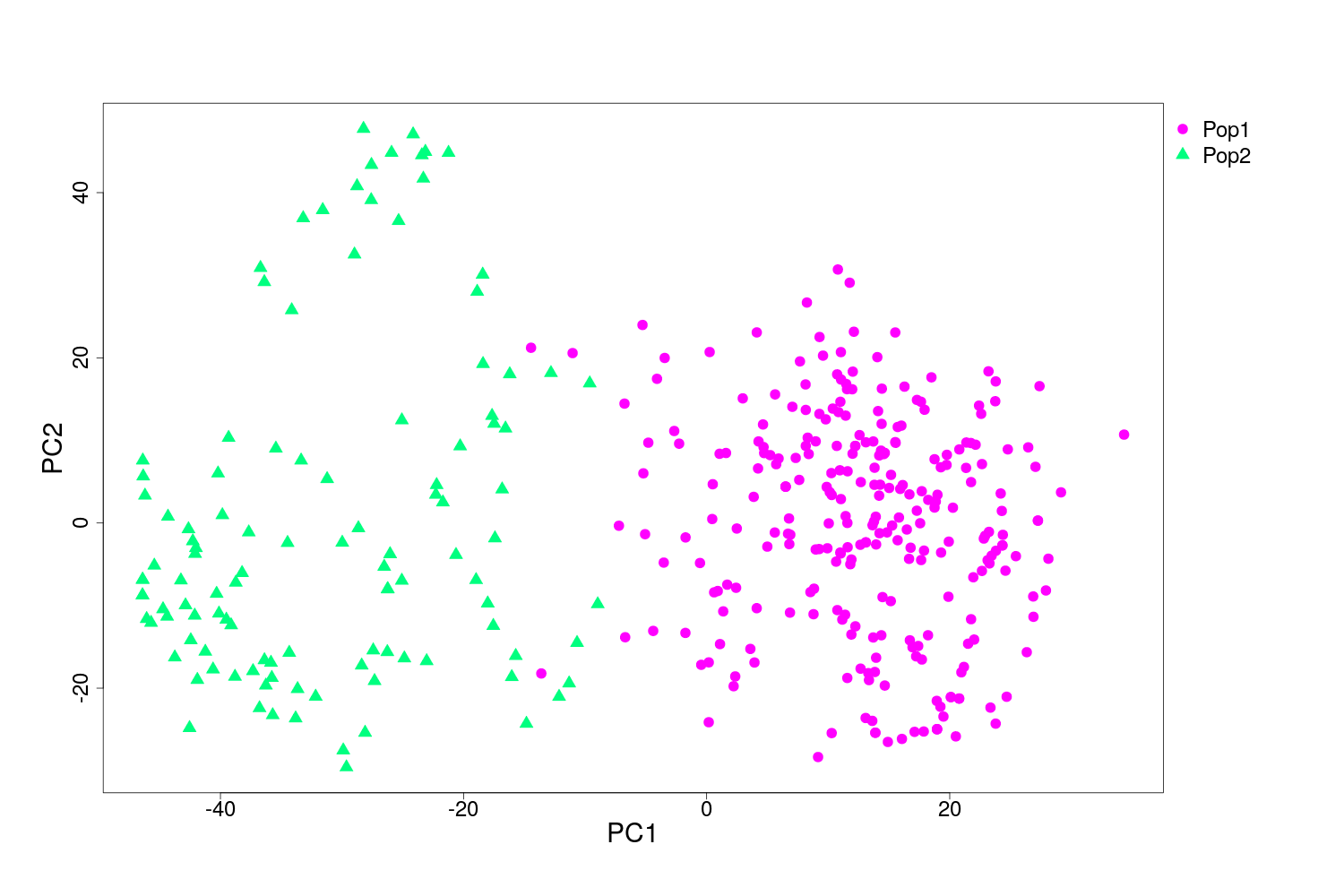

Supplement: FIGURE S6 — PCoA analysis with 16 core SSRs set. Pop1 and Pop2 are labeled in pink and green, respectively. [file Image_6.JPEG]

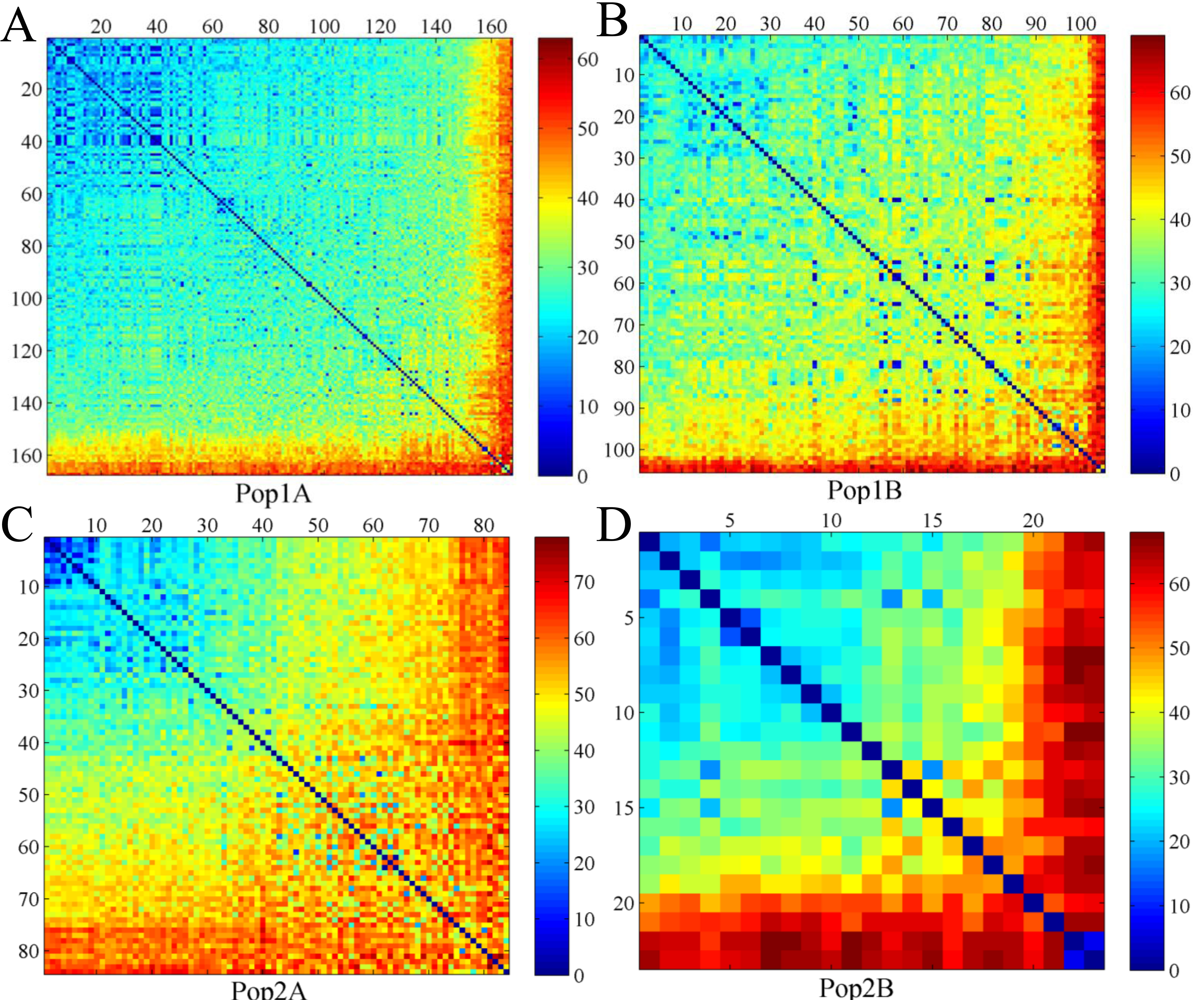

Supplement: FIGURE S7 — Heatmap of pairwise comparison matrix derive from differential SSR genotypes in Pop1A (A), Pop1B (B), Pop2A (C), and Pop2B (D). Red to Blue indicated the increasing differential SSR genotypes. [file Image_7.JPEG]
